# The reference genome sequence of the scarlet follicle, *Sterculia lanceolata*, reveals a paleo-polyploidization and its impact on fruit quality and fruit dehiscence

**DOI:** 10.1007/s10142-026-01829-9

**Published:** 2026-02-16

**Authors:** Youtao Hu, Youpeng Zhang, Hongbin Zhang, Jiahao Zhang, Guilian Guo, Kejing Yang, Bing-Yan Shao, Jia-Yu Xue, Robert Henry, Wenquan Wang, Fei Chen

**Affiliations:** 1 National Key Laboratory for Tropical Crop Breeding, College of breeding and multiplication (Sanya Institute of Breeding and Multiplication), Hainan University, Sanya, 572025 China; 2 College of Horticulture, Bioinformatics Center, Academy for Advanced Interdisciplinary Studies, Nanjing Agricultural University, Nanjing, 210095 China; 3 Queensland Alliance for Agriculture and Food Innovation, The University of Queensland, St Lucia, Brisbane, Australia

**Keywords:** Sterculia lanceolata, Malvaceae, Whole-genome duplication (WGD), Karyotype evolution, Seed development, Fruit dehiscence, Comparative genomics

## Abstract

*Sterculia lanceolata*, a tree species of the Malvaceae family with notable ornamental and medicinal value, has long been constrained in genetic research and breeding applications due to the lack of genomic resources. In this study, we report for the first time a high-quality, chromosome-level genome assembly of this species, aimed at elucidating its evolutionary history and the genetic basis of key traits. We constructed the genome using PacBio HiFi sequencing and further assembled it into 20 pseudochromosomes with the aid of Hi-C technology, yielding a final genome assembly size of 602.8 Mb with a contig N50 of 29.3 Mb and a BUSCO completeness of 98.7%. The assembly includes the identification of 20 pseudochromosomes and the annotation of 35,873 protein-coding genes, with an annotation rate of 96.4%. By integrating genomic data from other Malvaceae species, we analyzed the karyotype evolution of *S. lanceolata* and revealed the basal ploidy level of the family. Comparative genomic analyses uncovered significant syntenic relationships and whole-genome duplication (WGD) events among Malvaceae species, thereby clarifying the trajectory of karyotype evolution. Moreover, the study identified key regulatory gene families associated with fruit dehiscence (homologs of *SHP1/2*, *FUL*, *IND*, and *ALC*) that have undergone extensive expansion in *S. lanceolata* as a consequence of ancient polyploidy events. The reference genome provided in this study not only serves as a critical resource for evolutionary research in Malvaceae but also establishes a foundational framework for molecular breeding, genetic improvement, and conservation of *S. lanceolata* and related species.

## Introduction

*Sterculia lanceolata*, an important member of the genus *Sterculia* within the family Malvaceae, is a striking subtropical evergreen tree (Ma et al. 2016). It is renowned for its distinctive economic value and long-standing traditional medicinal uses (Prasad et al. 2022), with a broad geographic distribution primarily across subtropical regions of Asia, spanning southern China, Vietnam, Thailand, India, and beyond (Bawri et al. 2024). The species exhibits remarkable adaptability to the humid and variable climatic conditions characteristic of these regions. In addition to serving as a major regional source of timber, its roots, leaves, and seeds have been widely utilized in traditional medicine (Van Sam et al. 2008). They have been commonly applied to treat symptoms such as pain, swelling, and bruising caused by trauma or blood stasis. Previous studies have shown that plants of the genus *Sterculia* are generally rich in flavonoids and their derivatives, compounds that have been demonstrated to possess diverse biological activities, including antibacterial, anti-inflammatory, antioxidant, and anticancer properties (Wang et al. 2024). Within forest ecosystems, *S. lanceolata* also plays an essential role in providing both food sources and habitats for a variety of organisms. Despite its pronounced ecological and economic importance, our understanding of its genetics remains extremely limited. To date, the NCBI public database contains only a single transcriptome dataset derived from its leaves (PRJNA435648), and no complete nuclear genome has yet been reported for any member of the genus *Sterculia* (Eum et al. 2019). This knowledge gap has posed a significant barrier to uncovering the complex molecular mechanisms underlying its physiological characteristics, medicinal value, and ecological adaptability (Ganie et al. 2015).

This knowledge gap highlights the urgency of conducting in-depth genetic studies on *S. lanceolata*, as such research is essential to deciphering the genetic codes underlying its remarkable adaptability and economically important traits. The Malvaceae family also encompasses globally significant crops such as cotton (*Gossypium raimondii*) and durian (*Durio zibethinus*), and important ornamental plant species such as *Hibiscus* (Cvetković et al. 2021). In addition, recent chromosome-level genomes and metabolomic analyses of tropical woody and liana species such as *Ficus altissima* and *F. hirta*, the invasive vine *Merremia boisiana* and the ornamental tree *Spathodea campanulata* have highlighted how high-quality genomes can illuminate evolutionary divergence, specialized metabolite biosynthesis and ecological adaptation in tropical plants (Zhang et al. 2025; Wang et al. 2025; Guo et al. 2025). Although the genomes of multiple cotton and durian species have been decoded, providing profound insights into key traits such as fiber development and flavor compound biosynthesis, investigations into the evolutionary origins and genetic foundations of its important sister genus, *Sterculia*, remain in their infancy. More importantly, for certain lineage-specific biological traits within Malvaceae—such as the molecular mechanisms regulating precise fruit dehiscence following maturation—there is still a lack of in-depth cross-species comparative research (Pabón-Mora et al. 2014).

Therefore, this study aims to construct a high-quality, chromosome-level reference genome of *S. lanceolata*. By conducting an in-depth analysis of its genomic background and evolutionary history, we seek to identify the key gene families that determine its unique fruit morphology. Compared with existing studies on the genus *Sterculia*, which have been largely limited to morphological descriptions and transcriptome sequencing (Eom and Na 2019), this research provides, for the first time, a complete nuclear genome assembly, representing a major advancement. This will enable a deeper understanding of the molecular mechanisms underlying its ecological adaptability and valuable economic traits.

In addition, our findings bear direct significance for research across the entire Malvaceae family. Given the phylogenetic position of *S. lanceolata* within Malvaceae, our analysis of whole-genome duplication events and identification of key gene family expansions provide a valuable resource for comparative genomics. Specifically, the identification of critical genes associated with fruit dehiscence in *S. lanceolata* may offer novel genetic targets for the improvement of other related crops. Building on these insights, our study not only deepens the understanding of this economically important forest species but also provides essential scientific evidence for the evolution, genetic improvement, and conservation of Malvaceae.

## Materials and methods

### Experimental materials and sequencing technologies

Samples for sequencing of *S. lanceolata* were collected in the vicinity of the Innovation Research and Education Valley teaching area, Yazhou District, Sanya City, Hainan Province, China. DNA was extracted from young leaves, while samples for transcriptome analysis were obtained from various tissues of *S. lanceolata*, including roots, stems, functional leaves, and seeds. Samples were immediately flash-frozen in liquid nitrogen after collection and stored at − 80 °C in an ultra-low temperature freezer for subsequent nucleic acid extraction. Genomic DNA was randomly sheared, and short-read libraries of *S. lanceolata* were constructed following the TruSeq DNA sample preparation protocol. These libraries were then subjected to paired-end sequencing (150 bp read length) on the NovaSeq 6000 platform. In addition, a PacBio HiFi library was constructed. The library was prepared for sequencing using the PacBio Binding Kit (Wenger et al. 2019), in which primers and polymerases were ligated to the library. The final reaction products were purified with AMPure PB Beads and subsequently sequenced on the Revio sequencing system. Furthermore, Hi-C libraries were sequenced on the Illumina NovaSeq platform with paired-end reads of 150 bp. All transcriptome libraries were sequenced on the Illumina platform (Quail et al. 2008), generating paired-end reads of 150 bp. All sequencing data were processed using fastp v0.23.4 (Chen et al. 2018) to filter out low-quality reads and obtain clean data for downstream analyses.

### Genome size estimation based on k-mer analysis

A k-mer analysis was performed for genome survey. To obtain clean reads, the raw data were filtered by removing low-quality reads, short reads, adapter sequences, and polyG tails. Subsequently, KMC v3.2.4 (Kokot et al. 2017) was employed to conduct k-mer frequency distribution analysis with *k* = 19. Genome size, heterozygosity, and repeat content were estimated using GenomeScope v2.0 (Ranallo-Benavidez et al. 2020). The genome size of *S. lanceolata* was estimated to be 465.5 Mb, with a heterozygosity of 0.321%.

### Chromosome-level genome assembly

First, the raw HiFi data were converted into FASTQ format using samtools v1.21 (Li et al. 2009). To obtain clean HiFi and Hi-C data, fastp v0.23.4 (Chen et al. 2018) was applied to filter out low-quality reads, short reads, adapter sequences, and polyG tails from the raw datasets. The filtered HiFi data were then assembled into contigs using hifiasm 0.19.9 (Cheng et al. 2021) with default parameters. For chromosome-scale scaffolding, Hi-C data processing was performed using the ALLHiC v0.9.13 pipeline (Zhang et al. 2019). Clean Hi-C reads were first aligned to the assembled contigs to generate a Hi-C interaction matrix, and ALLHiC was then used to cluster contigs into linkage groups and to order and orient them within each group according to Hi-C contact frequency, thereby building chromosome-scale scaffolds. The scaffold organization was further refined with 3D de novo Assembly (3D-DNA, v180114) (Dudchenko et al. 2017), and potential mis-joins were manually inspected and corrected based on the Hi-C contact maps visualized in Juicebox v1.11.08 (Durand et al. 2016). To assess assembly quality, we conducted a comprehensive evaluation using BUSCO v5.1 (Seppey et al. 2019) with the eudicots_odb10 database to examine the presence and completeness of single-copy orthologs. Ultimately, we obtained a high-quality, well-assembled *S. lanceolata* genome sequence, providing a solid foundation for future functional studies.

### Gene prediction and annotation

We utilized RepeatModeler v2.0.3 (Flynn et al. 2020) to construct a *de novo* repeat library for the classification of repetitive sequences, and employed RepeatMasker v4.1.2 (Chen 2004) to identify them. In total, 376.7 Mb of repetitive elements were identified, constituting 59.6% of the *S. lanceolata* genome. All transcriptomic data were aligned to the genome using HISAT2 v2.2.1 (Kim et al. 2019). Subsequently, BRAKER v3.0.3 (Gabriel et al. 2024) was used to automatically train a species-specific parameter model and annotate gene structures, leveraging alignment evidence from both transcriptomic data and proteins. For functional annotation, the predicted protein-coding genes were aligned against the eggNOG database (Hernández-Plaza et al. 2023). This pipeline assigns orthology-based functional information, including Gene Ontology (GO) terms, KEGG Orthology (KO) identifiers, and functional categories to each query protein, providing a comprehensive functional description for the S. lanceolata gene set.

### Assembly and annotation of the chloroplast and mitochondrion genome

To assemble the chloroplast genome, we used the GetOrganelle v1.7.7.1 (Jin et al. 2020) software to assemble the Illumina paired-end sequencing data, specifying the -k option with values set to 21, 45, 65, 85, and 105, respectively. For mitochondrial genome assembly, we utilized the Oatk v1.0 (Zhou et al. 2025) toolkit for *de novo* assembly from PacBio HiFi sequencing reads, invoking the syncasm module within Oatk to filter specific k-mer sequences from the raw HiFi reads. We applied -k 1001 to set the minimum k-mer length and used -c 150 to set the minimum coverage threshold.

The resulting FASTA file was used as the input for annotation on the OGDRAW platform (Lohse et al. 2007) (https://chlorobox.mpimp-golm.mpg.de/OGDraw.html). Following the platform’s guidelines, we annotated the organellar genomes and chose the GenBank (GB) format for the output. To visualize the annotated genome, we accessed the “Upload” section of the OGDRAW online tool and submitted the GB file containing the *S. lanceolata* genome sequence. After selecting the appropriate options, we submitted the file for processing. OGDRAW then generated and displayed the annotated organellar genome in a graphical format, thus providing a clear representation of the organellar genetic structure.

### Synteny and whole-genome duplication analysis

We selected the protein sequence data of *H. littoralis*, *F. kwangsiensis*, *F. hainanensis*, and *S. lanceolata* as the basis for our collinearity analysis. To ensure the reliability of the results, we performed both intra- and inter-genomic self- and cross-comparisons using the BLASTP v2.12.0 tool, with an E-value threshold set to 1e-5. Subsequently, we utilized MCScanX v1.0.0 (Wang et al. 2012) to identify high-confidence collinear blocks. For the visualization of collinearity information, we used JCVI v1.5.1 (Tang et al. 2024), which effectively displays inter-chromosomal collinear regions. For the analysis of whole-genome duplication (WGD) events, we applied WGD v1.1.054 (Schranz et al. 2012) to calculate the WGD incidence rate and used the WGDI v0.6.5 (Sun et al. 2022) software to plot the *K*s value distribution, thereby estimating the timing and frequency of WGD events.

### Polyploidy and karyotype analysis

We pre-processed the protein data for *S. lanceolata*, *D. zibethinus*, *G. raimondii*, and *H. schizopetalus* using BLAST v2.9.0–2 (Altschul et al. 1997), selecting the output file format “-outfmt 6” and “-num_alignment 2”. Subsequently, we constructed the ancestral Malvaceae karyotype (AMK) using WGDI v0.6.5 (Sun et al. 2022) with the “-km” option. Based on the protein sequence information, we inferred the modern karyotypes of the studied species. To quantify the fission and fusion events that occurred in the evolutionary history of these species, we enumerated all homologous segments and calculated the corresponding fission and fusion counts.

### Identification and functional enrichment analysis of WGD genes

To identify genes in the *S. lanceolata* genome derived from whole-genome duplication (WGD), we first performed an all-versus-all BLASTP v2.12.0 (Lavigne et al. 2008) search of all protein sequences, setting an E-value threshold of 1e-5. Using these alignment results and gene location data, we detected collinear blocks and classified gene duplication types with the MCScanX v1.0.0 (Wang et al. 2012) toolkit. Subsequently, we conducted a Gene Ontology (GO) enrichment analysis on the identified set of WGD genes. We used the web-based tool agriGO v2.0 (Tian et al. 2017) (https://systemsbiology.cau.edu.cn/agriGOv2/) for the enrichment analysis. agriGO v2.0 performs gene enrichment using the Singular Enrichment Analysis (SEA) method, with the SEA parameters set as follows: Fisher’s exact test, Yekutieli correction (FDR under dependency), a significance level of 0.05, a minimum of 5 mapped genes, and analysis of the complete GO and molecular function. GO enrichment results were visualized as color-coded graphs and heatmap-like layouts using DataColor, which provides distinctive color mapping to highlight complex biological data relationships (He et al. 2024).

### Phylogenetic analysis and divergence time estimation

We collected protein data for 39 species from public databases such as TropiCODB (Dai et al. 2025) (https://bioinformatics.hainanu.edu.cn/tropdb/) and PLAZA 5.0 (Van Bel et al. 2022) (https://bioinformatics.psb.ugent.be/plaza/versions/plaza_v5_dicots/), focusing on plants from the Malvaceae family and the model plant *Arabidopsis thaliana*. Using OrthoFinder v2.5.5 (Emms and Kelly 2019), a tree-based method, we identified high-quality orthogroups among these species. The OrthoFinder output was optimized with TRIMAL (Capella-Gutiérrez et al. 2009), applying the -gt 0.6 option to remove short sequences within each orthogroup and using a -cons 60 threshold to retain only the most conserved sites. Subsequently, we performed a Maximum Likelihood (ML) analysis on the concatenated protein sequences of these species using the parallel version of RAxML v8.2.13 (Stamatakis 2014) (raxmlHPC-PTHREADS). This analysis aimed to infer the phylogenetic relationships in the context of *S. lanceolata*, thereby providing a clearer understanding of its evolutionary position relative to other plant species.

To estimate the divergence times among closely related species, including *H. littoralis*, *F. kwangsiensis*, *F. hainanensis*, *T. cacao*, *D. zibethinus*, *B. ceiba*, *G. hirsutum*, and *S. lanceolata*, we utilized the protein sequences of single-copy genes from Malvaceae plants. The divergence time estimation was conducted using the mcmctree v4.9 (Puttick 2019) tool in the PAML package. Following the analysis, the estimated divergence times were integrated into a phylogenetic tree reconstructed with IQTREE v2.4.0 (Minh et al. 2020), thereby providing a comprehensive understanding of the evolutionary relationships among the species.

### Identification and phylogeny of key fruit dehiscence genes

To elucidate the molecular regulatory mechanism of follicle dehiscence along the ventral suture in *S. lanceolata*, we first selected five known key regulatory genes from the model plant *Arabidopsis thaliana*: SHP1 (AT3G58780), SHP2 (AT2G42830), FUL (AT5G60910), IND (AT4G00120), and ALC (AT5G67110). The proteome data used in this study included those of *S. lanceolata*, *Gossypium hirsutum* (upland cotton), *Durio zibethinus* (durian), and *Arabidopsis thaliana*. Based on the conserved protein domain features of the target genes in *A. thaliana*, we downloaded the Hidden Markov Model (HMM) file for the MADS-box domain from the Pfam database v35.0 (Mistry et al. 2021). Using the hmmsearch program within the HMMER package v3.3.2 (Finn et al. 2011) with default parameters, a search was conducted against the combined proteomes of the four aforementioned species.

To elucidate the evolutionary relationships among the members of each gene family, we constructed phylogenetic trees for each family separately. First, a multiple sequence alignment of the homologous protein sequences for each family was performed using MAFFT v7.525 (Katoh and Standley 2013). Subsequently, based on the alignment results, phylogenetic trees were constructed using FastTree v2.1.11 (Price et al. 2010) with the WAG amino acid substitution model and the approximate maximum-likelihood method. Branch reliability was assessed based on Shimodaira-Hasegawa-like (SH-like) local support values. Finally, the resulting tree files were imported into the iTOL v6 (Letunic and Bork 2024) online platform for visualization and annotation.

## Results

### Chromosome-level genome assembly

**Fig. 1:**
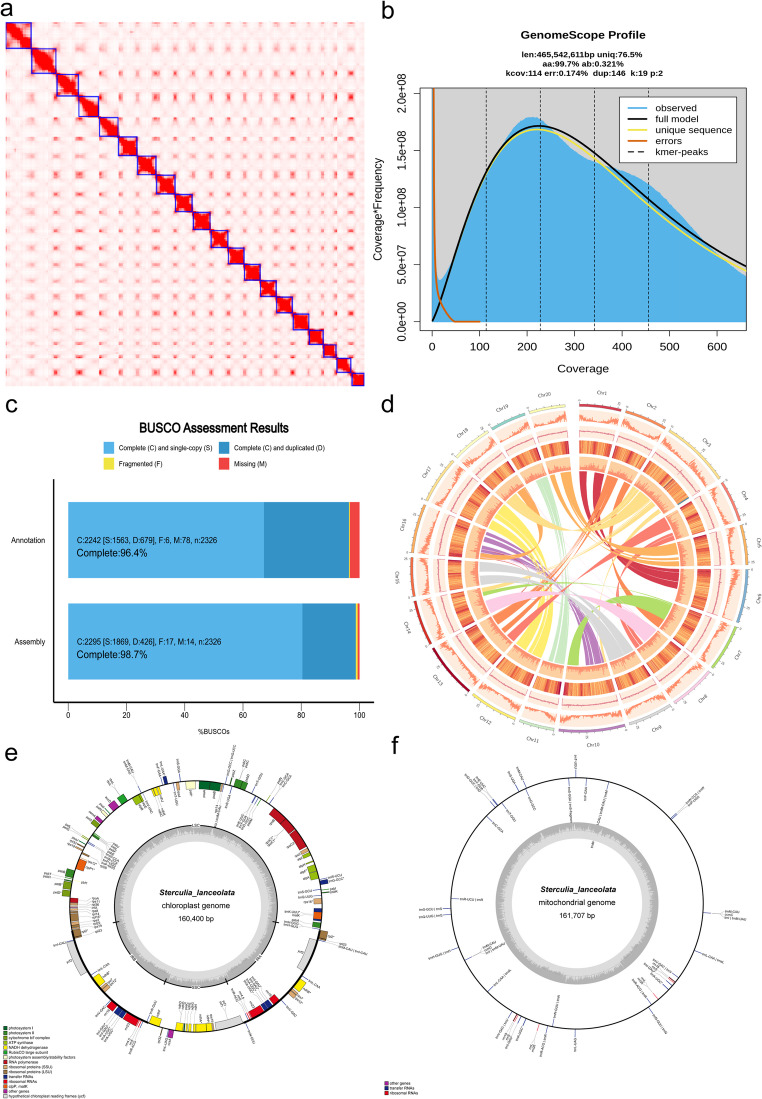
Genomic features of *S. lanceolata*. (**a**) Hi-C heatmap of the genome assembly. (**b**) Distribution of 19-bp short reads. (**c**) Assessment of completeness for the genome assembly and gene set annotation. (**d**) Genomic features of *S. lanceolata* (chromosome karyotype; gene density; GC content per Mb; repeat content per Mb; long terminal repeat (LTR) distribution; identification of syntenic regions among different chromosomes). (**e**) Chloroplast genome of *S. lanceolata*. (**f**) Mitochondrial genome of *S. lanceolata*

To gain a deeper understanding of the genetic characteristics and evolutionary history of *S. lanceolata*, we employed advanced sequencing technologies to successfully construct a high-quality, chromosome-level reference genome. The final assembly, corresponding to the primary (collapsed) haploid assembly generated by hifiasm, has a total size of 602.8 Mb with a contig N50 of 29.3 Mb, indicating excellent continuity. Using high-throughput chromosome conformation capture (Hi-C) technology, the vast majority of sequences were successfully anchored to 20 pseudochromosomes, representing the 2n = 40 diploid genome (Fig. 1a). The Hi-C interaction heatmap clearly displayed strong diagonal signals and very weak inter-chromosomal interactions, strongly supporting the high accuracy and completeness of our genome assembly at the chromosome level (Fig. 1a). To evaluate the overall quality of the genome, k-mer analysis was performed. The k-mer analysis indicated that the *S. lanceolata* genome has low heterozygosity (0.321%) (Fig. 1b). Such low heterozygosity reduces allelic divergence and makes accurate separation of haplotypes more challenging, but it facilitates the construction of a highly contiguous primary assembly, which provides a solid foundation for subsequent high-quality genome assembly, annotation, and downstream analyses.

Genome completeness is a core metric for evaluating its quality. We assessed the completeness of the genome using BUSCO v5.1 (Seppey et al. 2019). The results showed that the BUSCO completeness of the genome assembly reached 98.7% (Fig. 1c), indicating that nearly all core conserved genes were fully represented. This high score demonstrates that the *S. lanceolata* genome we obtained is a high-quality, highly complete reference genome, sufficient to support downstream analyses such as comparative genomics, evolutionary studies, and functional gene mining. In addition, we successfully assembled the complete chloroplast (160,400 bp) and mitochondrial (161,707 bp) genomes (Fig. 1e and f), providing additional layers of genetic information for species identification and phylogenetic research.

### Repeat sequence characterization and genome annotation

Following repeat annotation using both *de novo* prediction and homology-based approaches, we found that *S. lanceolata* contains a substantial proportion of repetitive sequences, accounting for 59.59% of the genome. Among these, 42.46% are retrotransposons and 3.59% are DNA transposons, with long terminal repeats (LTRs) comprising 40.94% of the genome. The overall genomic GC content reached 32.71% (Fig. 1d). By integrating *de novo* prediction, homology-based alignment, and transcriptome annotation, we identified a total of 35,873 protein-coding genes, with a BUSCO v5.1 completeness of 96.4% for the genome (Table 1). Notably, the number of genes in *S. lanceolata* exceeds that of closely related Malvaceae subfamily species, including *H. littoralis* (29,154 genes) (He et al. 2022)d *hainanensis* (34,388 genes) (Dong et al. 2025), suggesting that *S. lanceolata* may encode a broader repertoire of stress-resistance and developmental genes. This provides a valuable genetic resource for horticultural breeding research, with the potential to introduce novel genes to enhance resistance and growth-related traits.

**Table 1:** Genome features of *S. lanceolata*

Genome	S. lanceolata
Ploidy	2n = 40
Assembled genome size (Mb)	602.8
Genomic heterozygosity (%)	0.321
Contig N50 (Mb)	29.3
Number of contigs	188
Number of pseudochromosomes	20
Repeat sequence content (%)	59.59
GC content (%)	32.71
Number of gene models	35,873
Genome BUSCOs (%)	98.7
Gene BUSCO (%)	96.4

### Ancient Whole-Genome duplications shaping evolutionary dynamics

**Fig. 2:**
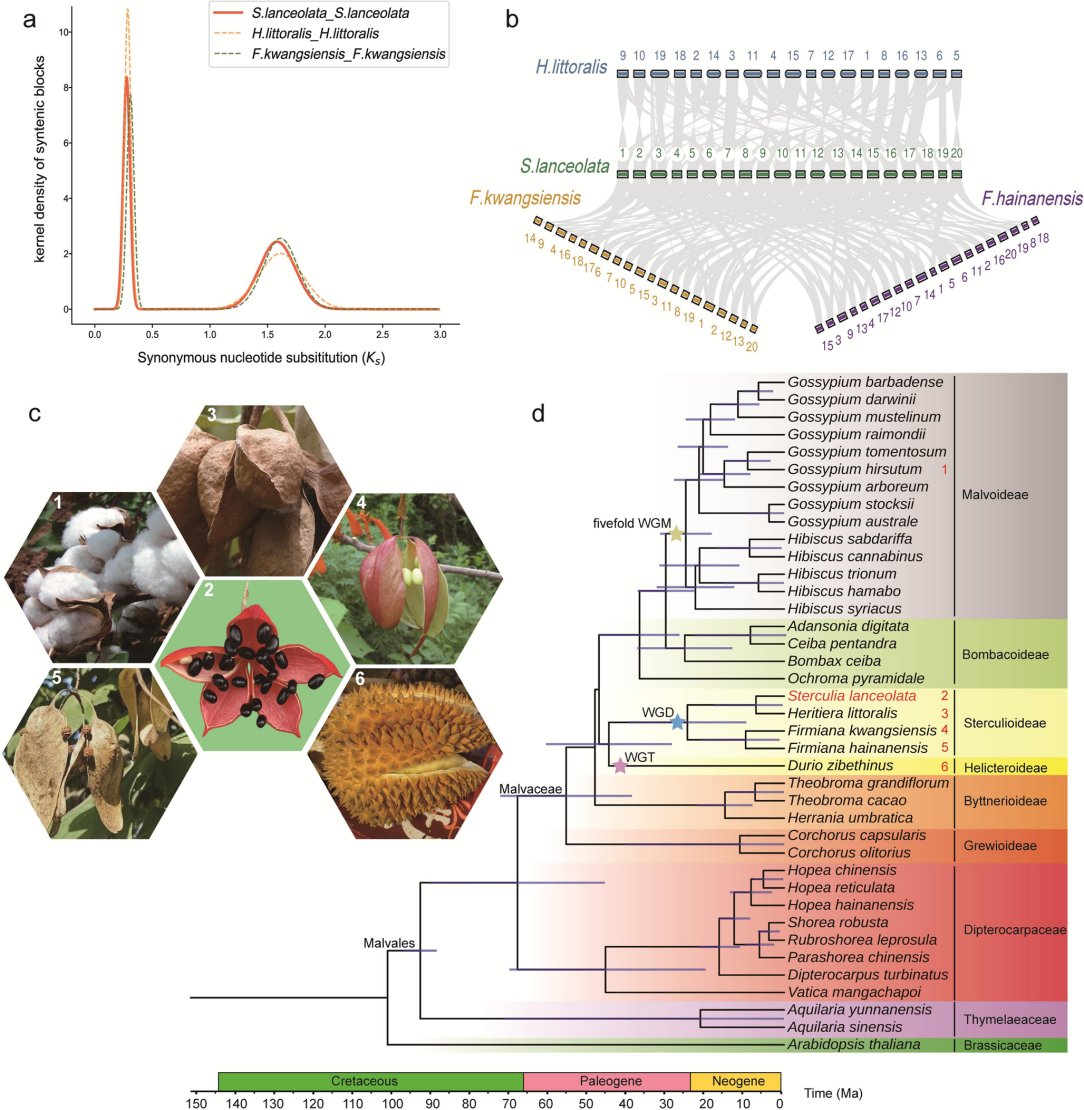
Genome evolution and phylogenetic analysis of representative Malvaceae species. (**a**) Distribution of synonymous substitution rates (*K*s) for paralogous genes within the genomes of three Malvaceae species. (**b**) Synteny between *S. lanceolata* and *H. littoralis*, *F. kwangsiensis*, and *F. hainanensis*. (**c**) Images of six Malvaceae species. (**d**) Phylogeny and divergence time estimation of 39 plant species

Whole-genome duplication (WGD) or polyploidy events are key drivers of plant evolution, providing abundant genetic material that facilitates adaptation and species diversification. To investigate the evolutionary history of *S. lanceolata* and related species, we constructed a phylogenetic tree encompassing multiple subfamilies within Malvaceae (Fig. 2d). The tree clearly places *S. lanceolata* within the subfamily Sterculioideae and reveals well-defined phylogenetic relationships with other Malvaceae species.

Notably, we identified several ancient polyploidy events at key nodes of the phylogenetic tree. For example, on the branch of Helicteroideae, which includes durian (*Durio zibethinus*), we detected a pronounced whole-genome triplication (WGT) event (Fig. 2d). This finding is consistent with previous studies and helps explain the large genome size and complex genetic background of durian.

To elucidate the polyploidy history and chromosomal evolution of Malvaceae plants, we conducted a comparative genomics analysis of *S. lanceolata* and its closely related species. Within the *S. lanceolata* genome, the distribution of synonymous substitution rates (*K*s) for paralogous gene pairs exhibits a pronounced peak at approximately 1.6, providing clear evidence for an ancient whole-genome duplication (WGD) event (Fig. 2a).

More importantly, analysis of *K*s distributions of paralogous gene pairs clarified the polyploidization history of *S. lanceolata*. The *K*s distribution shows a distinct peak at ~ 0.3 in *S. lanceolata* (Fig. 2a, red solid line). In combination with the WGD signals annotated on the species phylogeny (Fig. 2d), this pattern indicates a lineage-specific whole-genome duplication in *S. lanceolata*. In addition, an older *K*s peak at approximately 1.5–2.0 is shared among several Malvaceae species and corresponds to ancient polyploidy events at the family or deeper level. Inter-species synteny analysis (Fig. 2b) shows conserved collinear blocks and lineage-specific rearrangements among these genomes, which are consistent with the inferred WGD history.

### Chromosomal rearrangements shaped the unique 20 chromosomes of *S. lanceolata*

**Fig. 3:**
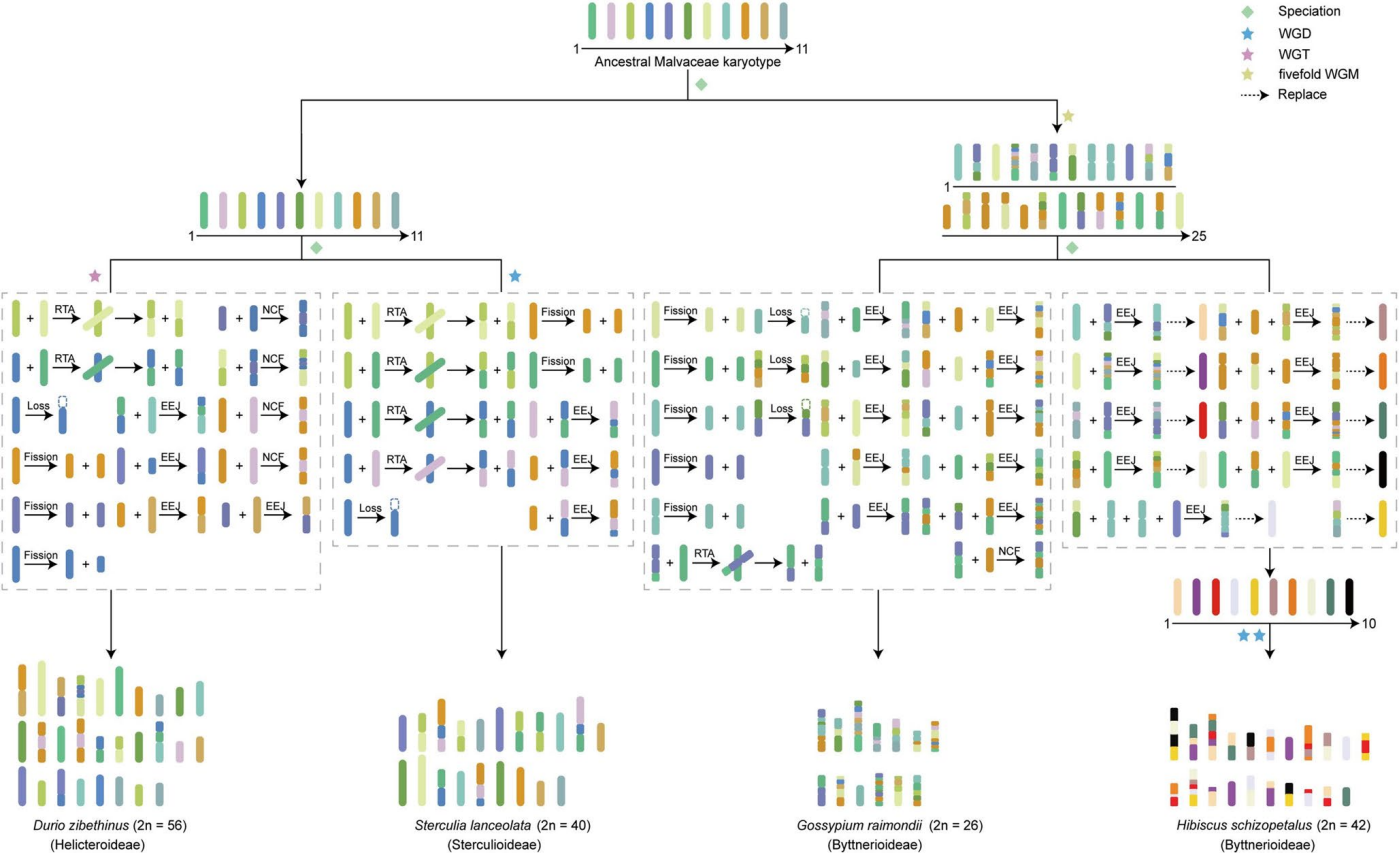
Karyotype analysis of *S. lanceolata* and three other Malvaceae species

Our reconstruction of karyotype evolution (Fig. 3) indicates that a common ancestor of Malvaceae possessed a karyotype with 11 chromosomes (*n* = 11). Following this ancestral stage, a major divergence occurred. The lineage leading to Malvoideae (including *Gossypium raimondii* and *Hibiscus schizopetalus*) underwent an ancient whole-genome pentaplication (WGM) event. In contrast, the lineage leading to *S. lanceolata* within Sterculioideae experienced an independent whole-genome duplication (WGD) event. Following this WGD, the *S. lanceolata* genome underwent extensive chromosomal rearrangements, including reciprocal translocations (RTA), end-to-end joining (EEJ), fissions, and losses, ultimately stabilizing into its current karyotype of 20 chromosome pairs (*n* = 20, 2n = 40).

For comparison, its close relative durian (*Durio zibethinus*, subfamily Helicteroideae) appears to have undergone another independent whole-genome triplication (WGT) event, followed by a series of complex fissions, nested chromosome fusions (NCFs), and other rearrangements, resulting in its modern karyotype of 2n = 56. *Gossypium raimondii* has maintained a relatively stable karyotype (2n = 26) with few subsequent changes, whereas *Hibiscus schizopetalus* experienced at least two additional WGD events, followed by extensive chromosomal fissions and fusions, culminating in its current karyotype of 2n = 42. These diverse evolutionary trajectories vividly illustrate the substantial differences in genome structural evolution among lineages following WGD events, and it is precisely these differences that have shaped the rich and varied biological characteristics of Malvaceae species.

### Dynamic changes in seed nutrient composition and gene accumulation driven by polyploidy

**Fig. 4:**
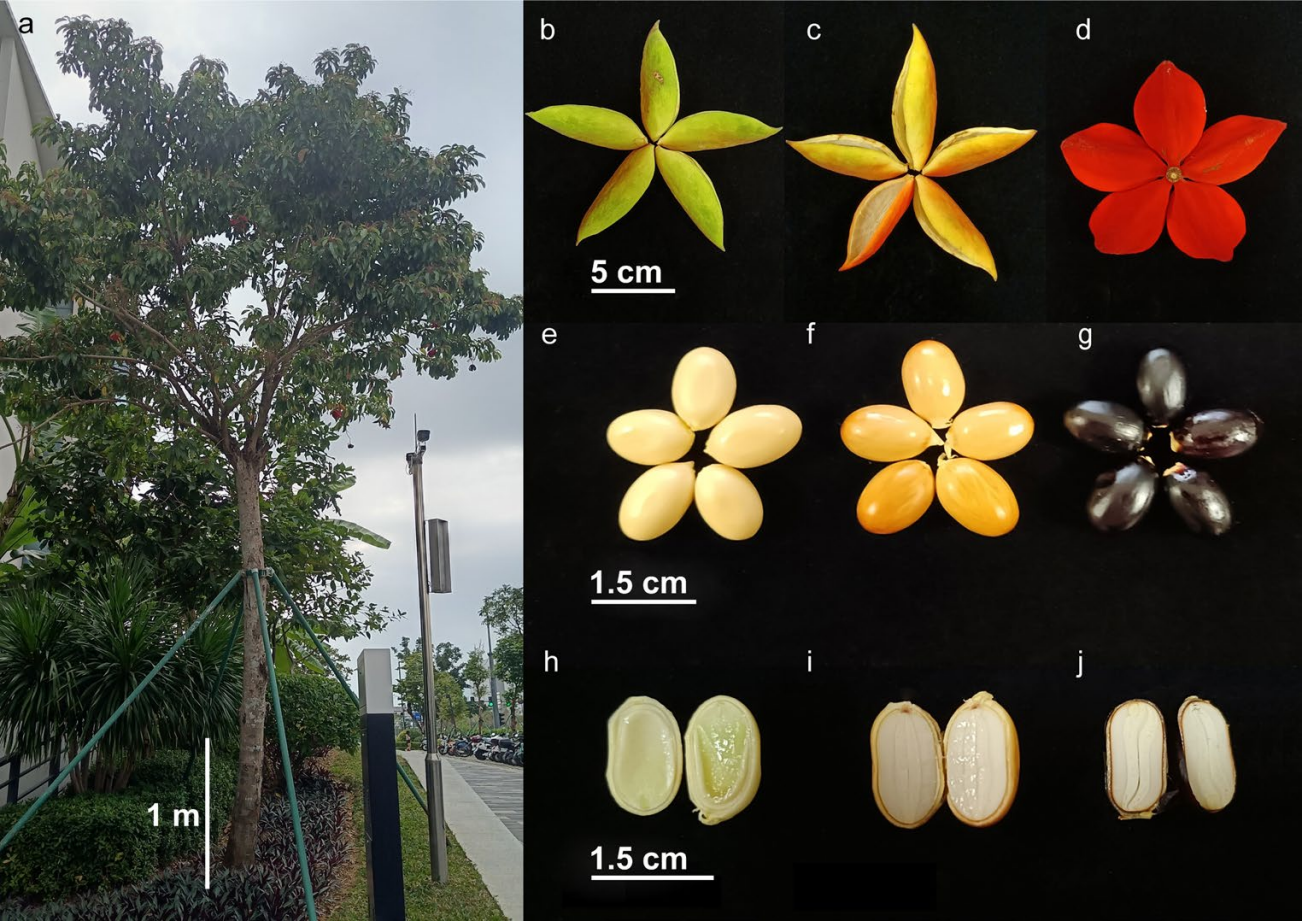
Morphological features of *S. lanceolata*. (**a**) Morphology of a mature plant. (**b**–**d**) Follicles (fruits) at different developmental stages. (**e**–**g**) Seeds at different developmental stages. (**h**–**j**) Longitudinal sections of seeds at various developmental stages

The seeds of *S. lanceolata* have attracted considerable attention due to their rich nutritional content and unique flavor. We conducted a systematic study of the morphology and nutritional composition of *S. lanceolata* seeds during their development from immature to mature stages (Fig. 4). Morphologically, both the fruit (follicle) and seeds undergo significant color changes throughout development (Figs. 4e–g).

**Fig. 5:**
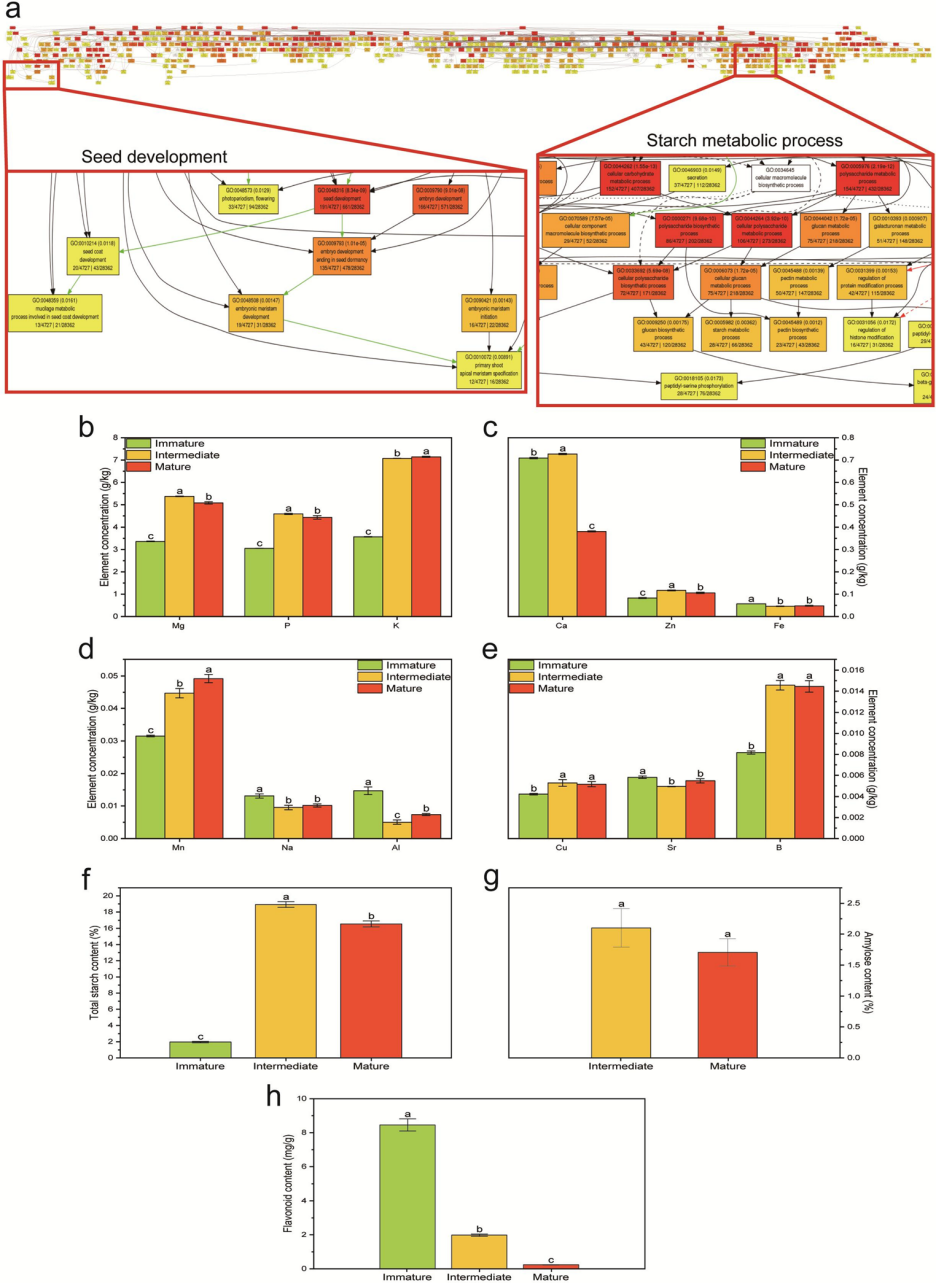
GO functional annotation and physiological–biochemical changes during seed development in *S. lanceolata*. (**a**) GO functional annotation of *S. lanceolata*, with seed development and starch metabolism highlighted within the red box. (**b**–**e**) Changes in mineral element contents in seeds at different developmental stages. (**f**) Changes in total starch content in seeds at various developmental stages. (**g**) Changes in amylose content in seeds at different developmental stages. (**h**) Changes in flavonoid content in seeds at different developmental stages

The results provided compelling evidence: among the WGD-retained genes, GO terms related to “Seed development” and “Starch metabolic process” were significantly enriched (Fig. 5a). This finding directly indicates that the WGD event in *S. lanceolata* did not randomly increase gene copy numbers, but rather led to a directed, large-scale expansion of gene repertoires associated with the synthesis and accumulation of seed nutrients. These expanded gene sets provide abundant raw material for the complex regulatory networks governing seed metabolism.

The metabolite profiling results provided direct evidence supporting this conclusion. We found that, as the seeds matured, the contents of several key mineral elements, including magnesium (Mg), phosphorus (P), and potassium (K), increased significantly (Fig. 5b), which is critical for energy storage in seeds and subsequent germination. Total starch content peaked during the mid-developmental stage and slightly declined at maturity, while amylose content also exhibited dynamic changes throughout seed development (Fig. 5f and g). Interestingly, we observed that flavonoid content was very high during the early stages of seed development but decreased sharply upon maturation (Fig. 5h). Flavonoids are generally associated with plant defense and bitterness, and their reduction is considered an evolutionary strategy to enhance seed palatability, attract animals for consumption, and facilitate dispersal (Cipollini and Levey 1997).

### Expansion and mechanistic study of gene families related to fruit dehiscence

**Fig. 6:**
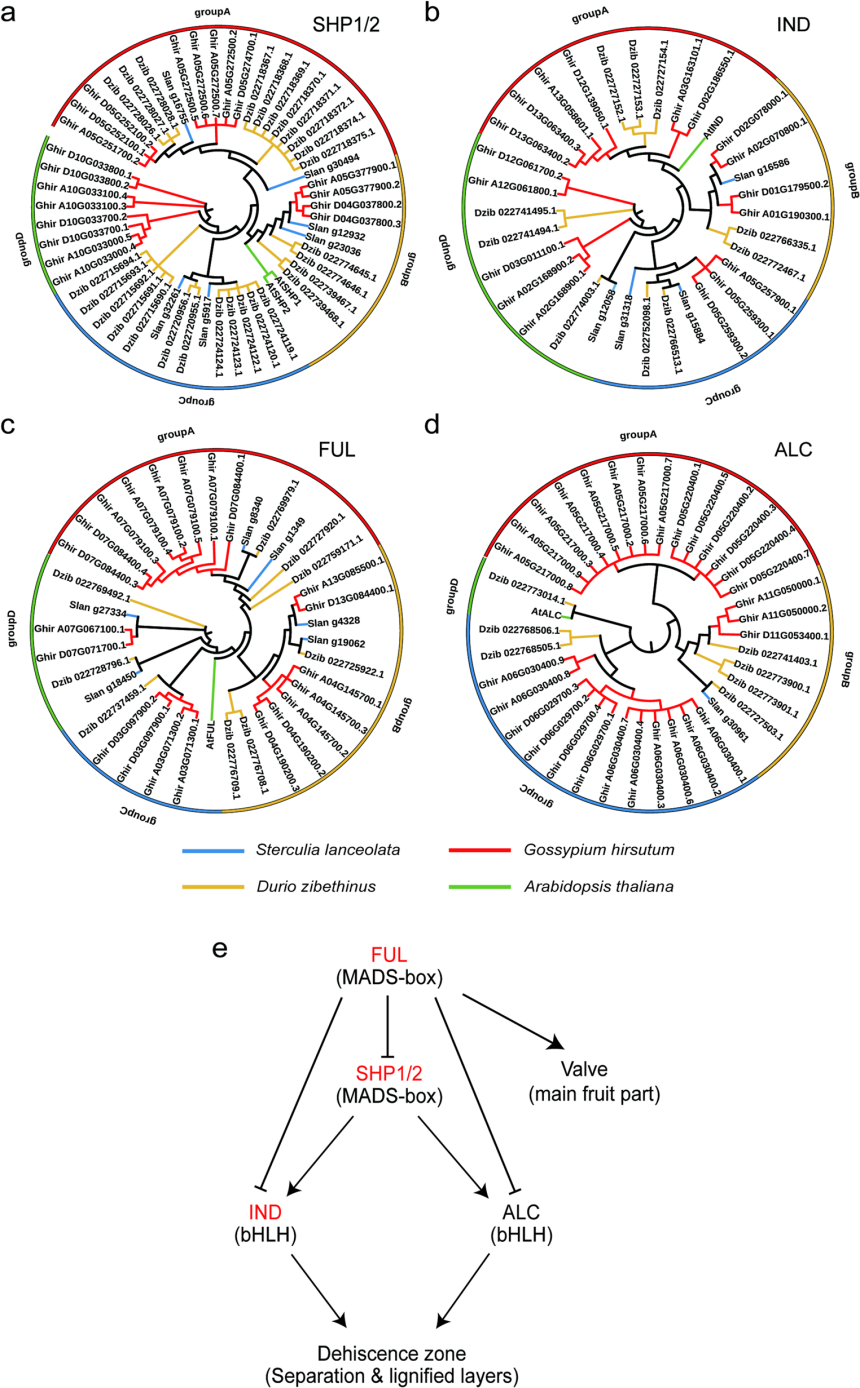
Phylogenetic relationships and regulatory pathway model of fruit dehiscence genes in *S. lanceolata*. (**a**–**d**) Maximum likelihood phylogenetic trees of the *SHP1/2*, *IND*, *FUL*, and *ALC* gene families. (**e**) Simplified model of the regulatory pathway controlling fruit dehiscence in *Arabidopsis thaliana*

To validate this model and investigate the unique dehiscence mechanism in *S. lanceolata*, we conducted a systematic phylogenetic analysis of these key gene families (Fig. 6a–d). In the analyses of the *SHP1/2*, *IND*, *FUL*, and *ALC* gene families, we observed that, compared with the model plant *Arabidopsis thaliana*, *S. lanceolata* (denoted as “Slan” in the figures) exhibits a notable expansion in gene copy numbers across most of these families (Figs. 6a–c). For instance, in the phylogenetic tree of the *SHP1/2* gene family, multiple “Slan” genes cluster together, forming species-specific gene clusters (Fig. 6a). In addition, Fig. 6e provides a schematic summary of the regulatory relationships among *FUL*, *SHP1/2*, *IND* and *ALC* in the fruit valves and dehiscence zone.

Fruit dehiscence is a critical developmental process governed by a finely tuned gene regulatory network that ensures a clear boundary between the valve and the dehiscence zone (Fig. 6e). Similar complex regulatory modules linking transcription factors to fruit development and ripening have also been reported in other tropical tree crops, such as diploid wax apple (*Syzygium samarangense*), where NAC genes were shown to regulate fruit development (Zhang et al. 2024). Within this regulatory network, the MADS-box transcription factors *SHP1/2* act upstream, activating the downstream bHLH factors *IND* and *ALC*. *IND* primarily orchestrates the specialization of the separation layer and lignified layer, working in concert with *ALC* to complete the formation of the separation layer, whereas loss of *ALC* typically results in dehiscence defects. In contrast, the MADS-box gene *FUL* plays a dominant role in valve growth and development, restricting differentiation of the dehiscence zone by antagonizing the *SHP1/2–IND/ALC* pathway. Through the activation by *SHP1/2* and the antagonistic action of *FUL*, this regulatory network collectively establishes the spatial boundary between the valve and valve margin, thereby enabling controlled dehiscence of mature fruits along a predetermined interface.

## Discussion

This study reports the first high-quality, chromosome-level reference genome for *S. lanceolata*, filling a critical gap in the genomics of the *Sterculia* genus within the Malvaceae family. This achievement not only establishes a solid genetic foundation for dissecting the species’ unique economic and medicinal traits but also provides a new and crucial phylogenetic node for resolving the complex evolutionary history of the entire Malvaceae family. The assembled genome exhibits excellent continuity (Contig N50 = 29.3 Mb) and high completeness (BUSCO = 98.7%), with a quality sufficient to support sophisticated comparative genomics and functional gene discovery. High-quality, chromosome-level assemblies of this type are increasingly recognized as indispensable resources for resolving plant genome structure, evolutionary history and trait-associated loci (Zhou et al. 2022). Furthermore, the genome’s low heterozygosity offers critical insights into the species’ reproductive biology, population history, and provides a crucial baseline for future conservation genetics. This research marks a pivotal transition for the study of the *Sterculia* genus, advancing it from traditional morphology and limited transcriptomics into the whole-genome era.

The whole-genome duplication (WGD) events revealed in our study are a core driving force in the evolution of *S. lanceolata* and the entire Malvaceae family. Through *K*s distribution analysis, we clearly identified two pivotal polyploidization events: an ancient WGD (*K*s ≈ 1.6) shared among Malvaceae species and a more recent, lineage-specific WGD (*K*s ≈ 0.3) within the *S. lanceolata* lineage. This recent WGD event is key to understanding the unique biological traits of *S. lanceolata*. Polyploidization provides abundant raw genetic material, enabling organisms to adapt to environmental changes and evolve novel functions, and ancient waves of polyploidization in flowering plants have been closely linked to facilitated adaptation to environmental stress (Wu et al. 2020; Zhang et al. 2020).

Compared to the whole-genome triplication (WGT) experienced by durian (*Durio zibethinus*) (Teh et al. 2017) and the pentaploidy (WGM) event in the cotton lineage (Wendel and Cronn 2002), the WGD in *S. lanceolata* demonstrates the diversity and complexity of polyploidization history within Malvaceae. Following a WGD, the genome typically undergoes drastic rearrangement and a process of diploidization. Our karyotype analysis reconstructed the evolutionary trajectory of *S. lanceolata* from a putative ancestral Malvaceae karyotype of *n* = 11. This pathway involved a WGD event (theoretically forming *n* = 22), followed by a series of complex chromosomal rearrangements including reciprocal translocations (RTA), fissions, and losses, which ultimately stabilized to form its characteristic *n* = 20 (2n = 40) karyotype. This model not only perfectly explains the origin of its modern chromosome number but also vividly illustrates the universal mechanisms of dynamic genome evolution post-polyploidization (Soltis et al. 2015).

One of the primary breakthroughs of this study is the direct link established between a macro-evolutionary WGD event and the species’ key economic traits. We innovatively identified the set of genes that originated from the recent WGD and were subsequently retained, then performed a functional enrichment analysis on this specific gene set. The analysis revealed significant enrichment in the categories of ‘seed development’ and ‘starch metabolic process.’

This finding provides strong evidence that gene retention post-WGD was non-random, instead favoring the selective expansion and preservation of genes related to nutrient accumulation in seeds. We then corroborated these genetic findings with metabolomic data, which demonstrated that as seeds mature, key mineral elements and total starch content increase significantly, while flavonoid compounds associated with astringency decrease sharply. Therefore, the observed changes in flavonoid content in *S. lanceolata* seeds likely reflect an evolutionary compromise balancing the dual demands of “self-protection” and “attracting dispersers” during co-evolution. This forms a complete evidentiary chain—from a genomic evolutionary event to functional gene set expansion, and ultimately to the formation of a desirable economic trait—profoundly uncovering the evolutionary underpinnings of the high nutritional value of *S. lanceolata* seeds. This research paradigm, which tightly integrates WGD with economic traits, remains relatively rare in plant genomics and is analogous both to research on the expansion of starch-related genes in potato (Van Harsselaar et al. 2017) and to work showing that an ancient WGD contributed to the diversification of flavor-related genes in the tea plant (*Camellia sinensis*) (Wang et al. 2021).

Similarly, the WGD event provides clues for elucidating another key biological trait in *S. lanceolata*: fruit dehiscence. The ability of a fruit to dehisce at the appropriate time is crucial for plant seed dispersal and propagation (Balanzà et al. 2016). In the classical Arabidopsis model, *FUL* is primarily expressed in the fruit valves and suppresses the formation of the dehiscence zone, whereas *SHP1/2*, *IND* and *ALC* act together to promote differentiation and lignification of the dehiscence zone, ultimately leading to fruit opening. Our study revealed that the core gene families regulating fruit dehiscence, including *SHP1/2*, *FUL*, *IND*, and *ALC*, all exhibit significant copy number expansion in the *S. lanceolata* genome. It is plausible that this WGD-driven increase in gene dosage led to a more complex and finely-tuned regulatory network. For instance, the expanded gene copies, potentially through subfunctionalization or neofunctionalization, may have enabled more precise control over the dehiscence zone and valve margins, thereby ensuring that the follicle dehisces efficiently and accurately along the ventral suture upon maturation (Lü et al. 2025). This discovery not only clarifies the developmental mechanisms in *S. lanceolata* but also offers potential homologous gene targets for the genetic improvement of other crops, such as preventing yield loss from pod shattering in rapeseed (*Brassica napus*) (Raman et al. 2014).

## Conclusions

This study reports the first successful construction of a high-quality, chromosome-level reference genome for *S. lanceolata*, revealing its genomic structure, gene composition, and evolutionary features. Through in-depth comparative genomic analysis, we elucidated that *S. lanceolata* underwent an independent, recent whole-genome duplication (WGD) event and have reconstructed in detail its evolutionary trajectory, which led to the formation of its current *n* = 20 karyotype via large-scale chromosomal rearrangements post-WGD.

Crucially, this study establishes a direct link between this macro-scale genomic evolutionary event and the species’ key agronomic traits. We demonstrate that the WGD-driven expansion of gene families directly resulted in the large-scale duplication and retention of key genes associated with ‘seed development,’ ‘starch metabolism,’ and ‘pod dehiscence.’ These changes at the genetic level are ultimately manifested at the phenotypic level as the rich accumulation of seed nutrients and a precise fruit dehiscence mechanism.

In conclusion, this research not only provides an invaluable resource for evolutionary and comparative genomics of the Malvaceae family but also establishes a solid molecular foundation for the genetic improvement, germplasm conservation, and exploitation of the medicinal value of *S. lanceolata*.

## Data Availability

The raw genomic sequencing data and transcriptomic reads have been deposited in the National Genomics Data Center under accession number PRJCA028606. The genome assembly, annotation files, and protein sequences are available at FigShare (10.6084/m9.figshare.30632720).
